# Requirements for hygienically safe, environmentally friendly dispensers for hand disinfectants and hand washing preparations

**DOI:** 10.3205/dgkh000337

**Published:** 2020-02-19

**Authors:** Axel Kramer

**Affiliations:** 1 Institute of Hygiene and Environmental Medicine, University Medicine Greifswald, Germany

**Keywords:** hand disinfectant, environmentally friendly, recyclable, biodegradable

## Abstract

**Aim:** Requirements make it practically impossible to refill the original container of hand disinfectants and hand washing preparations for dispensers that are fitted in doctors’ surgeries and hospitals. Therefore, dispensers are usually fitted with disposable containers. However, a number of disadvantages are associated with their use. The dispensers are often incompatible with refill containers from different manufacturers. The pump system and dispenser must be reprocessed after being fitted. Furthermore, the air suction of the pump can lead to internal contamination during operation. Therefore, proposals for developing a new generation of disposable containers are made.

**Methods:** Based on the recommendation from the *Commission for Hospital Hygiene and Infection Prevention* at the Robert Koch Institute, Berlin, regarding hand hygiene, the recommended requirements for disinfectant dispensers were analysed to propose how can they be implemented

**Results and discussion:** Flexible, transparent bags are proposed as disposable containers, based on the following advantages vs. rigid disposable containers. As the volume decreases, the bag shrinks correspondingly until it is completely empty. This means that the fill level is always clearly visible. Negative pressure can be applied to the bag. Caused by the negative pressure in the bag, the disinfectant is released onto the hand and a pump head is not required. The empty bag is disposed of using the outlet. This eliminates the need to reprocess both the pump head and the dispenser. Depending on the type of dispenser, the disinfectant can be removed manually or without coming into contact with it. Compared to a rigid dispenser system, material savings can be achieved if the new system is carefully designed. It is mandatory that the bag be made of recyclable or biodegradable material.

**Conclusion:** With the proposed technological solution, it is possible to create an alternative, hygienically safe, environmentally friendly dispenser for hand disinfectants and hand washing preparations.

## Zusammenfassung

**Zielsetzung:** Ein Nachfüllen von Händedesinfektionsmitteln und Handwaschpräparaten in das ursprüngliche Behältnis zur Beschickung von Spendern ist in Arztpraxen und in Krankenhäusern wegen der damit verbundenen Auflagen praktisch nicht möglich. Daher werden Spender üblicherweise mit Einmalgebinden bestückt. Deren Anwendung ist jedoch mit einer Reihe von Nachteilen verbunden. Häufig sind die Spender nicht mit Nachfüllgebinden unterschiedlicher Hersteller kompatibel. Das Pumpsystem und der Spender müssen nach der Beschickung aufbereitet werden. Außerdem kann es im laufenden Betrieb zur Innenkontamination über den Luftsog der Pumpe kommen. Daher werden Vorschläge zur Entwicklung einer neuen Generation von Einmalgebinden unterbreitet.

**Methode:** Anhand der Empfehlung der *Kommission für Krankenhaushygiene und Infektionsprävention* beim Robert Koch-Institut Berlin zur Händehygiene wurden die dort empfohlenen Anforderungen an Desinfektionsmittelspender analysiert, um Vorschläge zu deren Umsetzung abzuleiten.

**Ergebnisse und Diskussion:** Als Einmalgebinde werden flexible durchsichtige Beutel vorgeschlagen. Das ist mit folgenden Vorteilen gegenüber starren Einmalgebinden verbunden. Mit zunehmender Volumenabnahme schrumpft der Beutel bis zur vollständigen Entleerung. Damit ist der Füllstand jederzeit gut erkennbar. An den Beutel kann ein Unterdruck angelegt werden. Durch den Unterdruck im Beutel wird das Mittel auf die Hand freigegeben, und es kann auf einen Pumpkopf verzichtet werden. Der leere Beutel wird mit dem Auslass entsorgt. Dadurch entfällt die Aufbereitung sowohl des Pumpkopfs als auch des Spenders. Die Entnahme des Mittels kann abhängig vom Spender manuell oder berührungslos erfolgen. Bei umsichtiger Konstruktion ist im Vergleich zu einem starren Spendersystem eine Materialeinsparung erreichbar. Das Beutelmaterial muss recyclebar oder biologisch abbaubar sein.

**Schlussfolgerung:** Mit der vorgeschlagenen technologischen Lösung ist es möglich, einen hygienisch sicheren, umweltfreundlichen Spender wahlweise für Händedesinfektionsmittel und Handwaschpräparate zu schaffen.

## Introduction

Disinfectant dispensers are usually filled with disposable containers. Pharmacies and hospital pharmacies are legally permitted to refill these according to § 13 (2) paragraph 2 and 3 of the German Medicines Act (Arzneimittelgesetz/; AMG), but only within the scope of the supply mandate. Otherwise, the pharmacy requires a manufacturing authorisation according to § 13 AMG.

In doctors’ surgeries and in hospitals, manufacturing authorization is not required for the personnel to decant hand disinfectants if these are used exclusively within the facility. Since using these disinfectants within the facility does not mean they are placed on the market in any way, the filled disinfectant is not subject to the mandatory approval stipulated in § 21 AMG. However, according to § 67 (2) of the AMG, the competent supervisory authority must be notified of the refilling. A prerequisite for decanting is comprehensive quality assurance. This includes, among other things, cleaning and sterilising the disinfectant containers before refilling, decanting under aseptic conditions, and proper labelling that includes the date of decanting and expiry date as well as batch documentation [1], which would be very difficult to carry out in a doctor’s surgery. If a liability case arises, the reverse burden of proof is the sole responsibility of the person carrying out the work, i.e., they are liable for the quality of the decanted product. Product contamination, in connection with other hygiene failures, leads to a court ruling [2].

## Requirements or recommendations of the Commission for Hospital Hygiene and Infection Prevention (KRINKO) for dispensers for hand disinfectants and hand washing preparations

The overall objective is the prevention of microbial contamination when refilling the dispenser with a hand disinfectant or with a hand washing preparation by changing the disposable container without contaminating it, combined with the greatest possible ease of use. In the interest of sustainability, creating environmentally friendly solutions for dispensers is also important. In order to save costs, disposable containers should be universally applicable.

In alcoholic disinfectants, there is still a risk (albeit low) of contamination with spores [3], [4], as alcohols are not sporicidally active. The risk of contamination [5] is much higher for hand washing preparations, including antiseptic hand washing preparations, e.g., those with chlorhexidine digluconate as the active substance [6]. In the KRINKO recommendation [2], the following requirements and recommendations are made for dispensers of hand disinfectants and hand washing preparations regardless of the manufacturer and the type of dispenser:

- Equipped with non-refillable containers (disposable containers).
- Containers from different manufacturers can be used.
- Microbial contamination of the pump head is avoided during use.
- It is possible to check the filling level during operation.
- The date when the product was first used or the expiry date is documented.
- Disposable containers for both hand disinfectants and hand washing preparations for easier handling are recommended.
- Automatically operated dispenser systems are to be preferred due to the lower probability of contamination and transfer, as well as the positive influence they have on usage compliance because they are much easier to use.
- Oral intake from the unstable container should be difficult, thus making them suitable for psychiatric wards etc. as well as prisons.

Ideally, the dispenser should be approved for hand disinfectants as a medical device, because in this case the manufacturer is obliged to provide information on proper reprocessing in the package insert. As dispensers for hand washing preparations and creams are not considered medical devices, users must find out the reprocessing steps themselves. 

## Reflections on implementing the KRINKO recommendation

### Universal usability

In principal, one should be able to fit dispensers from different manufacturers into the disposable container for the hand disinfectants, hand washing preparations or creams, i.e., the disposable containers should be designed according to the same technological principle.

### Contamination-proof disposable container

A flexible, transparent bag that is impervious to pathogens on all sides is suggested as a disposable container. This has a number of advantages over rigid disposable containers, which will be described below. The more the volume decreases, the more the bag shrinks until it is completely empty. This means that the fill level is always clearly visible. Negative pressure can be applied to the airtight bag. This eliminates the need to document the date the product was first used or the expiry date. The negative pressure in the bag releases the disinfectant onto the hand and there is no need for a pump head. This eliminates the need for pump reprocessing, including changing the pump spring. The empty bag is disposed of with the outlet. This eliminates the need for reprocessing the dispenser. 

Since air does not have to be drawn in for transport, as is the case with the pump mechanism, but instead the disinfectant inside is released by the negative pressure, air cannot be a potential source of contamination. At the same time, due to the completely closed system, there is no change in concentration of alcohol-based hand disinfectants through evaporation.

### Operation

Depending on the dispenser, it should be possible to remove the disinfectant from the bag manually or without coming into contact with it.

### Ecological aspects

Material savings can be achieved compared to a rigid dispenser system if the new system is designed carefully. A biodegradable or at least recyclable plastic should be used for the disposable container. Due to the elimination of the pump system and the thinner wall thickness of a bag system, it can be assumed that the volume of waste is reduced compared to previous disposable containers. The fact that the pump head and dispenser do not need to be reprocessed is another ecological advantage. The fact that the disinfectant is dispensed using negative pressure ensures that the bag is completely emptied, which contributes to economical consumption.

### Other aspects to consider

Due to this kind of design, refilling is impossible. Theft for private use is not possible. 

When purchasing hand washing preparations and creams, check that the manufacturer has declared the product free of potentially pathogenic microorganisms, as primarily contaminated products can also present a risk of infection [7].

## Notes

### Competing interests

The author declares that he has no competing interests.
